# Quality over Quantity: Contribution of Urban Green Space to Neighborhood Satisfaction

**DOI:** 10.3390/ijerph14050535

**Published:** 2017-05-16

**Authors:** Yang Zhang, Agnes E. Van den Berg, Terry Van Dijk, Gerd Weitkamp

**Affiliations:** 1 Department of Spatial Planning & Environment, Faculty of Spatial Sciences, University of Groningen, P.O. Box 800, 9700 AV Groningen, The Netherlands; t.van.dijk@rug.nl; 2 Department of Cultural Geography, Faculty of Spatial Sciences, University of Groningen, P.O. Box 800, 9700 AV Groningen, The Netherlands; a.e.van.den.berg@rug.nl (A.E.V.d.B.); s.g.weitkamp@rug.nl (G.W.)

**Keywords:** urban green spaces, health, green space availability, neighborhood satisfaction, quality of life, happiness

## Abstract

There is increasing evidence that the quality of green space significantly contributes to neighborhood satisfaction and well-being, independent of the mere amount of green space. In this paper, we examined residents’ perceptions of the quality and beneficial affordances of green space in relation to objectively assessed accessibility and usability. We used data from a survey in two neighborhoods (*N* = 223) of a medium-sized city in the Netherlands, which were similar in the amount of green space and other physical and socio-demographic characteristics, but differed in the availability of accessible and usable green spaces. Results show that residents of the neighborhood with a higher availability of accessible and usable green spaces were more satisfied with their neighborhood. This difference was statistically mediated by the higher level of perceived green space quality. Neighborhood satisfaction was significantly positively related to well-being. However, residents of the two neighborhoods did not differ in self-reported well-being and beneficial affordances of green space. These analyses contribute to a further understanding of how the accessibility and usability of green spaces may increase people’s neighborhood satisfaction. It highlights the importance of perceived quality in addition to the amount of green space when examining the beneficial effects of green space.

## 1. Introduction

In recent decades, considerable evidence has accumulated suggesting that green space in the living environment may positively contribute to the overall quality of life of urban residents. Particularly, the quantity or amount of green space in the living environment has been associated with health and well-being benefits (e.g., see for reviews, [1,2]). However, the quality of green spaces, in terms of features, for example, accessibility and usability, may also play a role [3,4,5]. The importance of quality over quantity was convincingly demonstrated in a recent quasi-experimental study in two neighborhoods in the Dutch city of Groningen [6]. The two neighborhoods differ in expert-assessed accessibility and usability of green space, but were matched for amount of green space and other relevant physical and socio-demographic characteristics. Despite the similarity in the amount of green space, residents of the neighborhood with more accessible and usable green spaces reported more attachment to the neighborhood green space and better mental health. The present paper builds on these previous findings by examining residents’ perceptions of the quality of the green spaces in the same two Groningen neighborhoods in relation to neighborhood satisfaction and well-being.

Quality of green space has thus far mostly been measured with expert assessments, such as checklists, in situ observations, and Geographical Information System (GIS) analyses. A disadvantage of expert-determined green space quality is that it does not take into account the appraisals of laypersons about their own environment. Following their daily experiences, laypersons have acquired first-hand knowledge of their neighborhood, and may be more qualified than experts to assess the qualities of green spaces [7]. In general, green space perception can be considered an example of environmental perception as an established approach to investigate how people assess their environment. It can provide insight into the characteristics of the environment that contribute to quality of life. Measuring people’s perceptions also has applied value, in terms of monitoring the quality of environments and supporting environmental decisions [8]. It is thus important to assess people’s perceptions of green space quality in addition to objective (expert) assessments.

There is no golden standard to measure perceived green space quality. Previous studies have often used single questions such as the general perceived quality of green space (e.g., [9,10]). A few studies have used more elaborate questions to measure perceived green space quality in estimating its impacts on health and well-being. For example, an Australian study used five questions to capture the attributes of perceived neighborhood greenness [11]. Results indicate that higher perceived greenness of a neighborhood resulted in higher physical and mental health scores. In a recent study in Turkey, respondents were asked to evaluate various aspects of perceived green space quality in relation to physical activity and health [3]. Results show that providing nearby green spaces that are perceived as large and visible as well as clean and well-maintained may be an effective strategy to improve physical activity and people’s health.

Most of the indicators of green space quality have thus far pertained to use, such as accessibility, maintenance, perceived safety, presence of amenities or absence of litter [12]. Recently, a broader set of green space quality indicators have begun to be acknowledged and studied, including perceived restorative potential and other beneficial affordances. The theoretical rationale behind these studies is that people’s perceptions of the beneficial affordances of green space for their health and well-being may guide their positive reactions to green space [13,14]. In line with these notions, an experimental study showed that greater preferences for simulated natural vs. built environments were statistically mediated by the greater mood-improving potential of natural scenes [14].

In terms of outcome variables, beneficial effects of green space have thus far mostly been measured with general measures of self-reported (mental) health (e.g., [15,16]) and well-being (e.g., [17,18]). However, increasing attention is being given to the importance of green spaces for people’s relationships with the places in which they live (e.g., [19,20]). In particular, several studies have demonstrated the importance of green space for neighborhood satisfaction [7,21,22]. For example, a survey conducted in two urban neighborhoods in Belgium showed that residents of the greener neighborhood were more satisfied with their neighborhood, and happier with their lives [22]. Among several environmental and social neighborhood qualities asked about, perceptions of neighborhood green space were found to be the most important predictor of neighborhood satisfaction and happiness. Differences in the objective quality of green space may also indirectly influence neighborhood satisfaction through residents’ perception and evaluation of these qualities. For example, an analysis of 725 neighborhoods in central Ohio showed that neighborhood satisfaction was indirectly related to the physically assessed vegetation rate through residents’ perceptions of the greenness of their neighborhoods [7].

In the present study, we examined residents’ perceptions of the quality and beneficial affordances of green spaces in relation to neighborhood satisfaction and well-being. We used data from a survey in two neighborhoods of Groningen, a medium-sized city in the Netherlands, which were similar in the amount of green space, but differed in expert-rated accessibility and usability of the green spaces. We predicted that residents of the neighborhood with more accessible and usable green spaces are more satisfied with their neighborhood and happier with their lives. We also predicted that residents of the neighborhood with more accessible and usable green spaces perceive the green space in their neighborhood as higher in quality and more beneficial to their well-being. Finally, we explored the possible mediational roles of perceived green space quality and perceived green space affordances in differences in satisfaction and well-being between the neighborhoods.

## 2. Methods

### 2.1. Study Design

**Figure 1:**
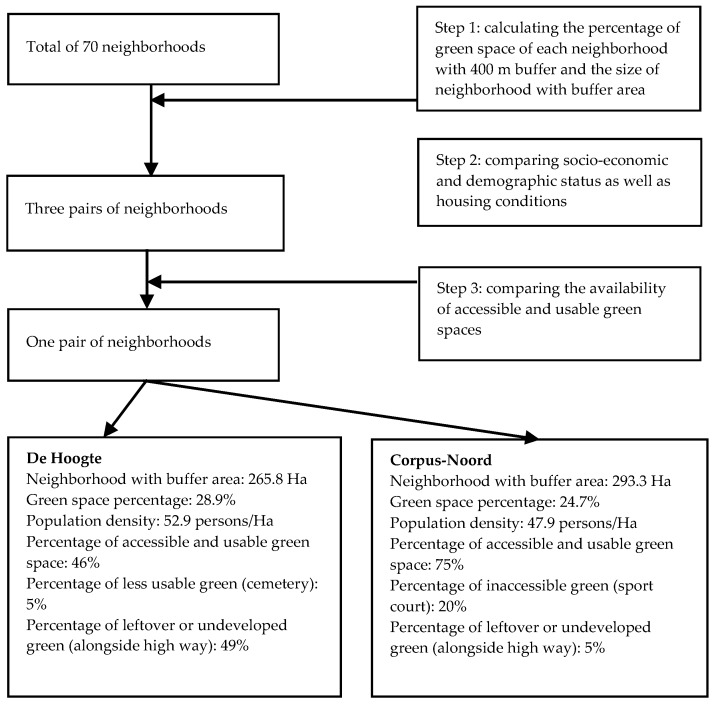
Summary of the procedure of neighborhood selection (for more details see [6]).

The data were derived from a study in two neighborhoods described by Zhang et al. [6]. Most importantly, in this study, we followed a stepwise procedure to select two urban neighborhoods in the city of Groningen that were comparable in green space quantity and socio-economic and demographic status, but contrasting in the availability of accessible and usable green spaces [22,23,24]. This selection procedure is summarized in Figure 1. One pair of neighborhoods was found to best meet the requirements, which are De Hoogte and Corpus Den Hoorn-Noord (Corpus-Noord).

Within the set of all neighborhoods, De Hoogte and Corpus-Noord were relatively similar in socio-demographic composition. However, there were some notable differences. Among other things, De Hoogte contained more low income households and more rental houses, which suggests that De Hoogte was of slightly lower socio-economic status than Corpus-Noord. De Hoogte also had a somewhat higher percentage of men, as well as more residents in younger age categories. The latter difference could imply more social interaction among young families with children in De Hoogte, which may impact residents’ perceptions of their neighborhood.

Based on GIS analyses and field observations, it was determined that De Hoogte has a low availability of green spaces that are fully accessible and usable (46%) while Corpus-Noord has a high availability of fully accessible and usable green spaces (75%). As shown in Figure 1, most of the inaccessible and unusable green space in De Hoogte consists of leftover or undeveloped green spaces alongside a highway (49%). In addition, there are two cemeteries (5%) in De Hoogte which aim at specific group users and lack usability for most neighborhood residents. Corpus-Noord has sport courts (20%) that are not publicly accessible for neighborhood residents, and some leftover spaces along the highway (5%).

### 2.2. Questionnaire and Measures

Data were collected by means of paper-mailed questionnaires that were randomly distributed in the two neighborhoods in June 2014. The questionnaire included questions about the socio-demographic background of the respondents (e.g., age, gender, income level, etc.), questions about the green spaces in the neighborhood, and questions about neighborhood satisfaction and other general quality-of-life indicators. The four main measures selected for the present analyses were neighborhood satisfaction, well-being, perceived green space quality and perceived green space affordances. In the data collection, we guaranteed that the answers of participants would be anonymized and only used for academic research. This study was approved by the Research Ethical Committee of the Faculty of Spatial Sciences, University of Groningen (registration number: 201703).

Neighborhood satisfaction was measured by a single-item question that asked respondents to indicate how satisfied they are with their neighborhood on a five-point scale ranging from ‘very dissatisfied’ to ‘very satisfied’ (e.g., [7,22]).

Well-being was measured by a single item that asked respondents to rate their current level of happiness on an 11-point scale with 0 = extremely unhappy and 10 = extremely happy (e.g., [25]).

Perceived green space quality was measured with a six-item scale that was similar to scales used in previous studies [19,26,27]. Respondents were asked to evaluate the provision of six use aspects including facilities, amenities, natural features, incivilities, accessibility, and maintenance, using a five-point scale ranging from 1 = strongly disagree to 5 = strongly agree. The scale showed sufficient reliability, Cronbach’s α = 0.78.

Perceived beneficial affordances were measured with a self-developed four-item scale that asked respondents to rate the extent to which the green spaces in their neighborhood promote quality of life, health, recreation, and social interaction, again using a five-point scale with 1 = strongly disagree and 5 = strongly agree. The scale showed good reliability, Cronbach’s alpha = 0.86.

### 2.3. Sample

In total, 276 out of 2750 distributed questionnaires were returned. Of these, 223 contained (almost) complete data available for analysis (net response rate 8.1%). Occasional missing values (<1%) were imputed using the average scores of the non-missing items. The net responses were 90 in De Hoogte and 133 in Corpus-Noord. The survey data confirmed that the sample was representative for the socio-demographic profiles of the two neighborhoods as derived from the census data, with the exception that female respondents were overrepresented in De Hoogte. However, due to this overrepresentation the two samples turned out to be better matched in gender distribution (61.1% females in De Hoogte vs. 55.6% Corpus-Noord) than our selection procedure indicated.

The survey data further confirmed the differences in age and socio-economic status as already indicated by the census data. Residents of De Hoogte were on average about 10 years younger (*M* = 39 years) than residents of Corpus-Noord (*M* = 49.6 years), with a concomitant shorter length of residence (*M* = 8.3 years vs. *M* = 13.1 years). Furthermore, there were more households with a net low income (<1000 euro per month) in De Hoogte (27.8%) than in Corpus-Noord (17.3%). For more details on the descriptive statistics of the sample, see Zhang et al. [6].

### 2.4. Data Analysis

Statistical analyses were performed using SPSS version 20 (IBM, Armonk, NY, USA). All differences between the two neighborhoods were tested using simple one-way ANOVAs with neighborhood as the independent variable. Mediation analyses were carried out using a linear regression analysis following procedures described by the mediation analysis of Baron & Kenny and MacKinnon et al. [28,29]. The Monte Carlo method was used to calculate the 95% confidence interval (CI) for the indirect effects [30]. Since the two samples differed in age, length of residence, and income, these variables were included as covariates in all analyses. Preliminary analyses of the relations between these covariates and the four outcome variables revealed that age was significantly positively correlated with neighborhood satisfaction (*r* = 0.14), while household income was significantly positively correlated with neighborhood satisfaction (*r* = 0.18) and overall well-being (*r* = 0.30). Length of residence was not significantly related to any of the outcome variables.

## 3. Results

### 3.1. Neighborhood Satisfaction and Overall Well-Being

Consistent with our predictions, residents of Corpus-Noord, with a high availability of accessible and usable green spaces, were significantly more satisfied with their neighborhood than residents of De Hoogte, with a low availability of accessible and usable green spaces (see Table 1 for an overview of the mean adjusted values in the two neighborhoods, controlled for age, length of residence, and income). Contrary to the expectations, there were no significant differences in happiness between the two neighborhoods. Neighborhood satisfaction was, however, significantly related to happiness, *r* = 0.33, *p* < 0.001, which supports the relevance of neighborhood satisfaction as a place-based component of overall well-being.

### 3.2. Perceived Quality and Beneficial Affordances of Green Spaces

Consistent with our predictions, residents of Corpus Noord perceived the green spaces in their neighborhood as higher in quality than residents of De Hoogte. Inspection of the adjusted means for the six individual items of the scale shows that this higher perceived quality applied to the broad range of measured use aspects, but with a somewhat lesser extent to maintenance. Residents of Corpus-Noord did not perceive their neighborhood green spaces as more beneficial on any of the four aspects measured (quality of life, health, recreational use, social interactions). This latter finding implies that residents’ perceptions of the beneficial affordances of green space do not qualify as a mediator of the differences in neighborhood satisfaction.

### 3.3. Mediation Analysis

**Figure 2:**
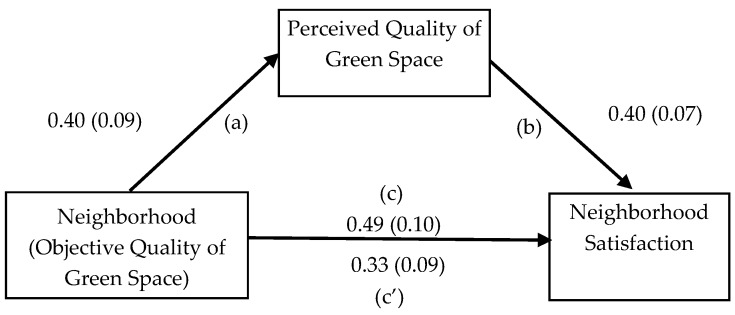
Mediation model showing the effects of neighborhood (0 = De Hoogte, 1 = Corpus-Noord) green space on neighborhood satisfaction, as mediated by the average perceived quality of green spaces. All relationships were estimated with age, length of residence, and income as covariates. Unstandardized regression weights are shown, with standard errors between parentheses.

Basic conditions for mediation of neighborhood differences were met for neighborhood satisfaction as a dependent variable and perceived green space quality as a mediator. As shown in Figure 2, neighborhood (0 = De Hoogte, 1 = Corpus-Noord) was significantly positively associated with neighborhood satisfaction, *b* = 0.49, *p* < 0.001 (path c). Second, neighborhood was significantly associated with perceived quality of green space, *b* = 0.40, *p* < 0.001 (path a). Third, perceived quality of green space was significantly associated with neighborhood satisfaction, while controlling for neighborhood, *b* = 0.40, *p* < 0.001 (path b). Fourth, the estimate for the difference in neighborhood satisfaction was reduced by 33% when estimated while controlling for perceived quality of green space (path c’). The statistical significance of the mediation effect (0.16) was confirmed by the confidence interval for the indirect (mediated) effect, which did not include zero, 95% CI = 0.13 to 0.19.

## 4. Discussion

This paper explored differences in neighborhood satisfaction and overall well-being between two neighborhoods that are to a large extent comparable in the amount of green space, socio-economic and demographic status, and other neighborhood environment characteristics, but differ in the availability of accessible and usable green spaces. We used mediational analysis to assess the role of perceived quality and beneficial affordances of green space in these differences. Results showed that residents of the neighborhood with more accessible and usable green spaces were more satisfied with their neighborhood, and that this difference was statistically mediated by differences in perceived (use) quality of the green spaces.

### 4.1. Perceived Green Space Quality and Beneficial Affordances of Green Space

Our findings suggest that the objectively-assessed availability of accessible and usable green spaces aligned with residents’ perceptions of the quality of the green spaces in their neighborhood in terms of use characteristics (facilities, amenities, natural features, incivilities, accessibility, and maintenance). Previous studies have reported mixed results regarding the agreement between objective and subjective measures of green space [31]. The consistency between the objective and subjective measures found in the present study could be due to the fact that we combined field observations with GIS analyses for our objective analyses of green space quality, whereas other studies have often used only one measurement approach. It is also possible that our measure of perceived green space quality, which comprised six use aspects, was able to better capture the complex construct of green space quality than simpler single-item questions. In general, the results suggest that objective, expert-based assessments of green space quality can be a good proximate of residents’ subjective perceptions. This is important, because expert-based quality assessments are typically less costly and time-consuming than surveys.

The neighborhood with higher objective and subjective green space quality was not perceived to provide more beneficial affordances to health, quality of life, recreation and social interaction. We were also unable to detect any differences between the neighborhoods in self-reported well-being. However, in previous analyses we did demonstrate significant differences between the neighborhoods in mental health [6]. It is therefore conceivable that these null-findings reflect methodological limitations arising from the use of a non-validated scale which may not capture all relevant beneficial affordances of green space, and the use of a single-item measure to assess well-being. It could also be that residents are not well capable of judging the beneficial effects of green spaces. This latter interpretation is in line with previous analyses suggesting that people in general tend to underestimate the beneficial effects of nearby nature [32]. It also fits with the finding of the present study that neighborhood satisfaction (which was related to green space quality) is positively correlated with happiness (see also [33]), suggesting a more indirect effect of environmental features on well-being [34].

### 4.2. Neighborhood Satisfaction and Well-Being

The finding that residents feel more satisfied with their neighborhood when they perceive the green spaces in their neighborhood as higher in quality is consistent with previous studies (e.g., [7]). The present research adds to previous findings by showing that perceived green space quality is related to objectively-assessed accessibility and usability of green spaces, independent of the amount of green space. This further strengthens the case for the importance of green space quality, in addition to amount of green space. The present study also shows that differences in satisfaction between neighborhoods that were selected for their objectively assessed difference in accessibility and usability of green spaces were statistically mediated by the perceived quality of the green spaces. This finding confirms the mediating role of residents’ perceptions of green space in relationships between objective physical characteristics of green space and the subjective evaluation of one’s neighborhood.

Green space quality was not found to be an important predictor of well-being, measured in terms of happiness. However, as already noted above, the present findings support the idea that green space quality may indirectly promote general well-being by increasing neighborhood satisfaction.

### 4.3. Limitations and Recommendations for Future Study

The quasi-experimental design of our study in which we varied green space quality while keeping the quantity of green space constant allowed for a controlled analysis of the importance of green space quality. However, a limitation of our approach is that data on mediating and dependent variables were collected from the same source, which may cause concerns about common method bias. Moreover, only two neighborhoods were included, which may have limited the generalizability of the results to other populations and regions. Although the sample was generally representative for the two neighborhoods, the response rate (less than 10%) and number of respondents were quite low. This may be due to the use of a paper-mailed survey, in combination with the fact that participants did not receive any monetary compensation. Given the lack of information on the respondents’ interactions with their neighborhood green spaces, we were also unable to take account of the effects of this variable. Another limitation is caused by the fact that we used self-developed measures for green space quality and beneficial affordances that are not well-validated. We also used a single-item measure to estimate self-reported neighborhood satisfaction and well-being, which may be less reliable than multi-item measures. The research was conducted in a cross-sectional setting, which limits the conclusions regarding a causal-inference [21]. For example, it is possible that people with higher well-being levels choose the neighborhood environment that they appreciate, which leads to higher levels of neighborhood satisfaction and perceived green space quality.

We suggest a few recommendations for future studies. First, to avoid same-source bias, future studies could use more objective information such as visits to the General Practitioner (GP) [35] or cortisol tests to measure well-being [36]. Second, it would be worthwhile to study relationships between neighborhood satisfaction, perceived green space quality and quantity in more large-scale epidemiological studies with large numbers of different types of neighborhoods. The response rate could be increased by using an online survey tool with follow-up reminders instead of paper-based questionnaires in combination with a monetary incentive. Third, future efforts are also warranted to obtain more detailed in situ observations of people’s interactions with actual green spaces in relation to the qualities and design of the green spaces and beneficial affordances. Fourth, more research is needed to validate our measures of green space quality and green space benefits. Multi-item measures on neighborhood satisfaction and well-being could also be used in future investigations. Lastly, our findings indicate that perceived green space quality is a mediator of the relationship between objectively-assessed green space quality and neighborhood satisfaction. To achieve a more conclusive statement, future research should further unravel these relationships, for example, in a longitudinal study.

## 5. Conclusions

In summary, this study shows that both the availability of accessible and usable green spaces and residents’ perceived quality of green spaces are significantly associated with neighborhood satisfaction, apart from the amount of green spaces. Thus, our findings suggest that studies on the amount of green spaces in relation to well-being outcomes need to be supplemented by studies that stress other dimensions of both objectively and subjectively measured green space quality. Moreover, perceived green space quality statistically mediated the link between the availability of accessible and usable green space and neighborhood satisfaction. This result suggests that, contrary to some previous findings, objective assessments of green space quality can be representative of residents’ subjective perceptions. Researchers and policy makers, therefore, need to pay attention to the quality of the neighborhood green spaces, which may be an important welfare enhancing approach.

## Figures and Tables

**Table 1:** Mean adjusted scores (with standard error between brackets) in the two neighborhoods, with the results of statistical analyses (controlled for age, length of residence and income).

Variable	De Hoogte (*n* = 90)	Corpus-Noord (*n* = 133)	*F*	*p*	*η_p_*^2^
**Neighborhood Satisfaction (1–5)**	3.39 (0.07)	3.88 (0.07)	26.12	<0.001	0.11
**Well-Being (Happiness; 0–10)**	7.24 (0.14)	7.34 (0.11)	0.34	0.563	0.00
**Green Space Quality (1–5; average score)**	3.21 (0.07)	3.61 (0.06)	20.04	<0.001	0.08
*Green spaces in my neighborhood*					
1. contain enough recreational facilities (e.g., play equipment, hard court, grass pitches for football).	3.10 (0.11)	3.39 (0.09)	4.13	0.043	0.02
2. provide amenities for sitting, picnic table, litter bins, signs and lighting in the night.	2.74 (0.11)	3.37 (0.09)	18.16	<0.001	0.08
3. have good natural features such as grass, trees and flower beds.	3.37 (0.09)	3.87 (0.07)	17.71	<0.001	0.08
4. are absent of incivilities (e.g., general litter, graffiti, dog mess, evidence of alcohol, drug use, broken glass and noise).	2.95 (0.11)	3.40 (0.09)	10.15	0.002	0.04
5. are easily accessed, there are many access points and enough walking paths, and roads around are not busy.	3.64 (0.08)	3.91 (0.06)	7.36	0.007	0.03
6. are well maintained.	3.47 (0.11)	3.73 (0.09)	3.46	0.06	0.02
**Beneficial Affordances of Green Space (1–5; average score)**	4.00 (0.08)	3.98 (0.06)	0.05	0.824	0.00
*Green spaces in my neighborhood*					
1. promote the quality of life.	4.12 (0.08)	4.16 (0.07)	0.08	0.779	0.00
2. promote health.	4.12 (0.08)	4.06 (0.07)	0.37	0.544	0.00
3. promote recreational use.	3.90 (0.10)	4.01 (0.08)	0.68	0.411	0.00
4. promote social interaction.	3.86 (0.10)	3.69 (0.08)	1.55	0.214	0.01
